# Intratumoral sustained release of resiquimod with ablative fractional laser induces efficacy in a cutaneous squamous cell carcinoma mouse model

**DOI:** 10.3389/fimmu.2025.1625867

**Published:** 2025-10-08

**Authors:** Martin Wiinberg, Fredrik Melander, Catharina M. Lerche, Thomas L. Andresen, Uffe H. Olesen, Merete Haedersdal

**Affiliations:** ^1^ Department of Health Technology, Technical University of Denmark, Kongens Lyngby, Denmark; ^2^ Department of Dermatology, Copenhagen University Hospital - Bispebjerg, Copenhagen, Denmark; ^3^ Department of Pharmacy, University of Copenhagen, Copenhagen, Denmark

**Keywords:** drug evaluation, preclinical, toll-like receptor agonists, delayed-action preparations, laser therapy, carcinoma, squamous cell

## Abstract

**Introduction:**

The Toll-like receptor (TLR) 7/8 agonist resiquimod has shown promise for precancerous lesions of cutaneous squamous cell carcinoma (cSCC) but remains unexplored as a treatment for cSCC. Additionally, ablative fractional laser (AFL) has been shown to enhance the efficacy of TLR7 agonist in mouse tumor models. This study investigates the efficacy of intratumoral resiquimod formulated into a sustained-release gel (RSQ-gel) in a cSCC mouse model and compares RSQ-gel with topical imiquimod (IMQ) cream, a clinically approved TLR7 agonist. We further examine whether adjuvant AFL enhances the efficacy of RSQ-gel.

**Methods:**

A syngeneic transplanted cSCC mouse model was established using cells from a UVR-induced cSCC mouse model. The immunostimulatory effects of RSQ-gel were assessed by analyzing the expression of the activation marker CD86 on plasmacytoid dendritic cells (pDC) and cross-presenting conventional type I dendritic cells (XCR1^+^ cDC1) via flow cytometry. Tumor growth and survival outcomes were evaluated for RSQ-gel as monotherapy and in combination with AFL.

**Results:**

RSQ-gel was associated with activation of pDCs and XCR1^+^ cDC1s in the tumor-draining lymph node, as indicated by higher expression of CD86 compared to IMQ (*P< 0.0001*, *P = 0.00175*, respectively). RSQ-gel monotherapy delayed tumor growth but did not prolong survival (*P = 0.0651*). However, combining RSQ-gel with AFL resulted in prolonged survival compared to AFL-treated and untreated mice *(P = 0.0153*, *P = 0.0214*, respectively). Weekly RSQ-gel treatment induced comparable efficacy to daily topical IMQ treatment.

**Discussion:**

RSQ-gel with AFL demonstrated significant antitumor efficacy in the cSCC mouse model. Local RSQ-gel combined with adjuvant AFL may offer a promising therapeutic approach for cSCC.

## 1 Introduction

Cutaneous squamous cell carcinoma (cSCC) is the second most prevalent type of epithelial cancer. Ultraviolet radiation (UVR) exposure is the primary risk factor for the development of cSCC. The standard of care is surgical excision, which results in high cure rates. However, cSCC lesions may develop in anatomically challenging locations or become locally invasive if not treated early, potentially rendering them ineligible for surgical excision (1). Locally applied drugs provide a feasible treatment alternative in inoperable cases of cSCC. Additionally, cSCC is considered a suitable candidate for immunotherapies due to its high mutational burden attributed to UVR exposure (2). This was highlighted with the approval of systemic treatment with the immune checkpoint inhibitor, cemiplimab, an antagonizing antibody targeting the programmed-death 1 protein, resulting in antitumor responses (2–6). Toll-like receptor (TLR) 7 agonists are another promising category of immunotherapeutic drugs for cSCC (7). However, topical administration of the TLR7 agonist imiquimod (IMQ) is currently only approved for the treatment of actinic keratosis, a precancerous form of cSCC, and shows limited efficacy in human cSCC (8–10).

TLR7 agonists trigger an innate immune response in plasmacytoid dendritic cells (pDC) characterized by the secretion of the pro-inflammatory cytokine, type I interferon-alpha (IFN-α) (11, 12). IFN-α secreted by pDCs activates conventional type I dendritic cells (cDC1), a subset of antigen cross-presenting cells characterized by their expression of X-C motif chemokine receptor 1 (XCR1). cDC1s are involved in mediating immunotherapy responses following TLR7 agonist treatment (13–17). TLR7 agonists enhance XCR1^+^ cDC1s’ ability to present antigens and activate cytotoxic T cells (12, 16–18), thereby inducing antitumor responses (19–22). Resiquimod, a derivative of IMQ, triggers both TLR7 and TLR8 and has been reported to induce a more potent immune response than IMQ (23–25). Topical resiquimod has demonstrated promising response rates against actinic keratosis in clinical trials but is yet be evaluated in the treatment of cSCC (26).

In this study, we formulated resiquimod into a sustained drug release matrix (RSQ-gel) that can be injected intratumorally, allowing resiquimod to be released over time at high concentrations while minimizing systemic drug spillover (27). The RSQ-gel is stored in a liquid phase, enabling administration with a conventional hypodermic needle, after which forms a semi-solid depot in the tumor.

Resiquimod is released over seven days from the RSQ-gel (27), thus requiring fewer treatments than topical creams. Additionally, the RSQ-gel consists of components that are biodegradable. A more comprehensive description of the RSQ-gel technology and its formulation is available in Jensen et al. (27). RSQ-gel has shown antitumor efficacy in the CT26 colon carcinoma cancer mouse model but has not been evaluated for the treatment of cSCC (27).

To evaluate the treatment potential of RSQ-gel, we generated a syngeneic transplanted cSCC mouse model based on the well-established spontaneous UVR-induced cSCC model (28). The transplanted cSCC model enables consistent and synchronized tumor growth, providing a robust model for evaluating therapeutic efficacy and biological responses under controlled conditions. In contrast, the parental spontaneous UVR-induced tumor model is highly resource-intensive as it requires larger group sizes to account for the unsynchronized development and growth of multiple tumors, making it more suitable for later-stage validation (29). The therapeutic potential of RSQ-gel is evaluated by examining its potential to activate pDCs and cDC1s within the tumor-draining lymph node (LN) and measuring tumor growth following weekly administrations. To enhance the potential antitumor efficacy of RSQ-gel, we combine RSQ-gel with adjuvant ablative fractional laser (AFL) treatment as preclinical studies of AFL have shown antitumor efficacy when combined with topical IMQ or immune checkpoint inhibitor treatment (29–34). The aim of this study is to investigate the therapeutic efficacy of intratumorally administered RSQ-gel in the transplanted cSCC model with and without AFL and compare the efficacy to daily administration of the clinically approved topical IMQ cream.

## 2 Materials and methods

### 2.1 Mice

Female C3.Cg-*Hr^hr^*/TifBomTac (Taconic, Ry, Denmark) immunocompetent mice were housed at Bispebjerg Hospital under a twelve-hour light/dark cycle at 24 °C with *ad libitum* feeding. Mice were acclimatized for at least one week prior to experiments. Mice included in studies were tattooed with identification numbers on their abdomen under sedation with 0.5 mL fentanyl citrate (0.158 mg/mL), fluanisone (5 mg/mL), and midazolam (2.5 mg/mL) since earmarking is not feasible in this mouse strain. All protocols and procedures were ethically reviewed and approved by the Danish Animal Experiments Inspectorate (permit number: 2019-15-0201-01666) and conducted in accordance with Directive 2010/63/EU.

### 2.2 Study design

**Figure 1:**
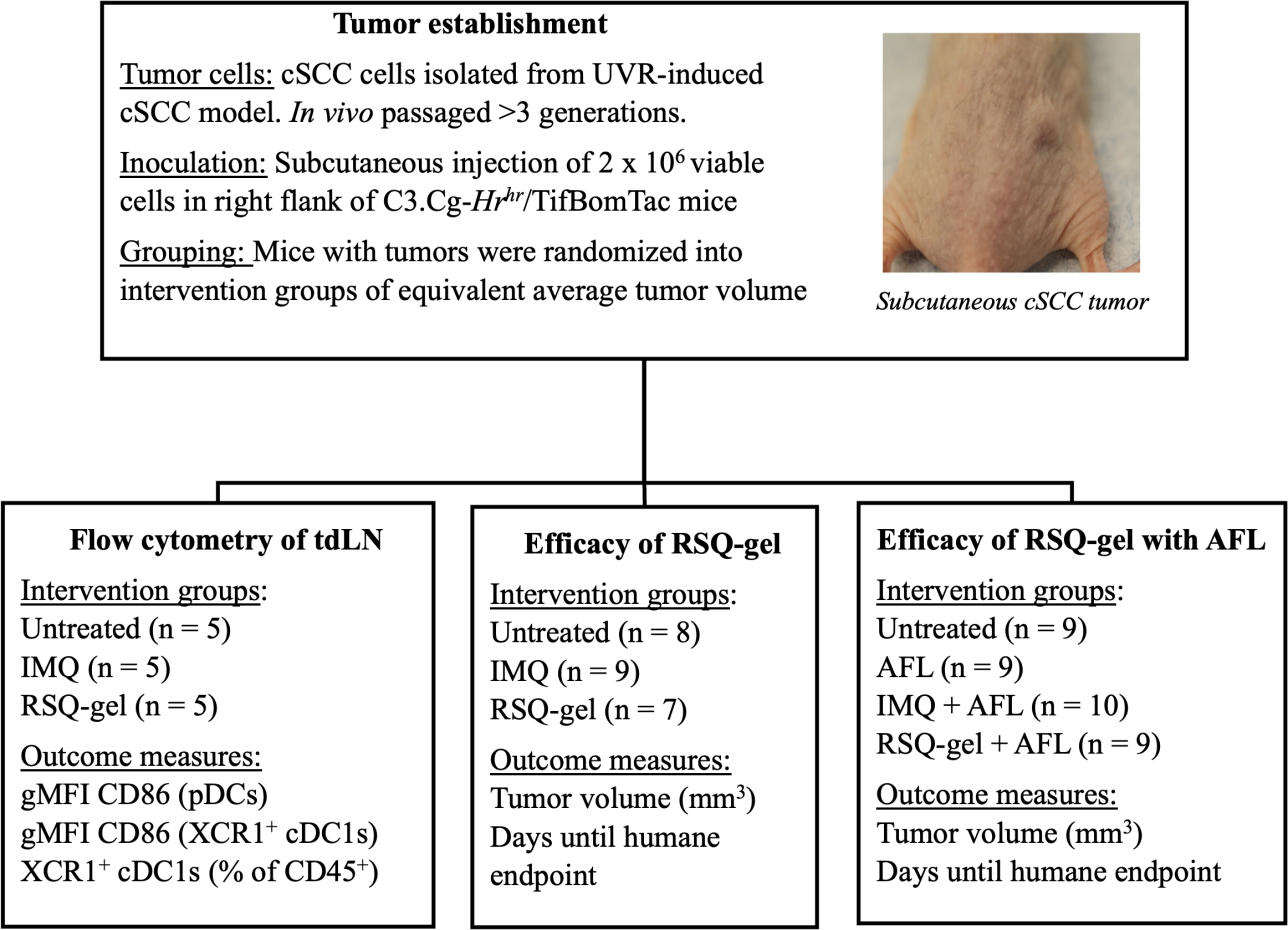
Experimental design. Schematic overview of study design. Tumors were established by subcutaneous injection of *in vivo* passaged UVR-induced cSCC cells into the right flank of hairless *C3.Cg-Hr^hr^/TifBomTac* mice. Mice were randomized into intervention groups with the same average tumor volumes. The study consists three experimental arms: (1) Flow cytometry of tumor-draining lymph nodes (tdLN), assessing CD86 activation marker on pDCs and XCR1^+^ cDC1s in untreated, IMQ, and RSQ-gel-treated groups,; (2) Efficacy of RSQ-gel, comparing untreated, IMQ, and RSQ-gel groups; (3) Efficacy of RSQ-gel with AFL, evaluating untreated, AFL, IMQ + AFL, and RSQ-gel + AFL groups. Abbreviations: AFL, ablative fractional laser; cDC1, conventional type I dendritic cells; cSCC, cutaneous squamous cell carcinoma; gMFI, geometric mean fluorescence intensity; IMQ, topical imiquimod cream; RSQ-gel, intratumoral injected sustained release formulated resiquimod gel; pDC, plasmacytoid dendritic cells; tdLN, tumor-draining lymph node; UVR, ultraviolet radiation; XCR1, X-C motif chemokine receptor 1.

The study was conducted in three parts: First, *ex vivo* flow cytometry analysis, measuring activation and recruitment of dendritic cells in tumor-draining LN following treatment, secondly, evaluation of efficacy of weekly RSQ-gel or daily IMQ monotherapy in a syngeneic transplanted cSCC tumor model, and lastly, assessment of the efficacy of weekly RSQ-gel or daily IMQ treatment with adjuvant AFL treatment in a syngeneic transplanted cSCC tumor model (**Figure 1**).

For flow cytometry, cSCC tumor-bearing mice were randomized and divided into three intervention groups with equal mean tumor volume of 140 mm^3^ (n = 5 per group): Untreated, IMQ or RSQ-gel. Treatment groups received a single treatment of either topical IMQ or intratumoral RSQ-gel. Mice were euthanized one day after treatment for *ex vivo* flow cytometry analysis of dendritic cells in the tumor-draining LN. Outcome measures were geometric mean fluorescent intensity (gMFI) of the activation marker CD86 on pDCs and XCR1^+^cDC1s as well as percentage of XCR1^+^cDC1s of all CD45^+^ immune cells in the tumor-draining LN.

To evaluate the treatment efficacy, mice in the monotherapy study were randomized into three groups with an average tumor size of 105 mm^3^: Untreated (n = 8), IMQ (n = 9), RSQ-gel (n = 7) and four groups in the combination study with an average tumor size of 129 mm^3^: Untreated (n = 9), AFL (n = 9), AFL+IMQ (n = 10), AFL+RSQ-gel (n = 9). Tumor dimensions were measured three times weekly with a digital caliper and tumor volume was calculated as Tumor volume = $\frac{Length\times Width^{2}}{2}$ . Humane endpoints were defined as a tumor volume >800 mm^3^ or weight loss >15% between measurements, failure to thrive or tumor ulceration. Mice with significant weight loss, failure to thrive or tumor ulceration were censored in survival analysis. Animal well-being was monitored daily throughout the studies. Outcomes measurements in survival studies were tumor volume and days until reaching humane endpoint. Investigators were not blinded during the described studies.

### 2.3 Tumor model establishment

**Figure 2:**
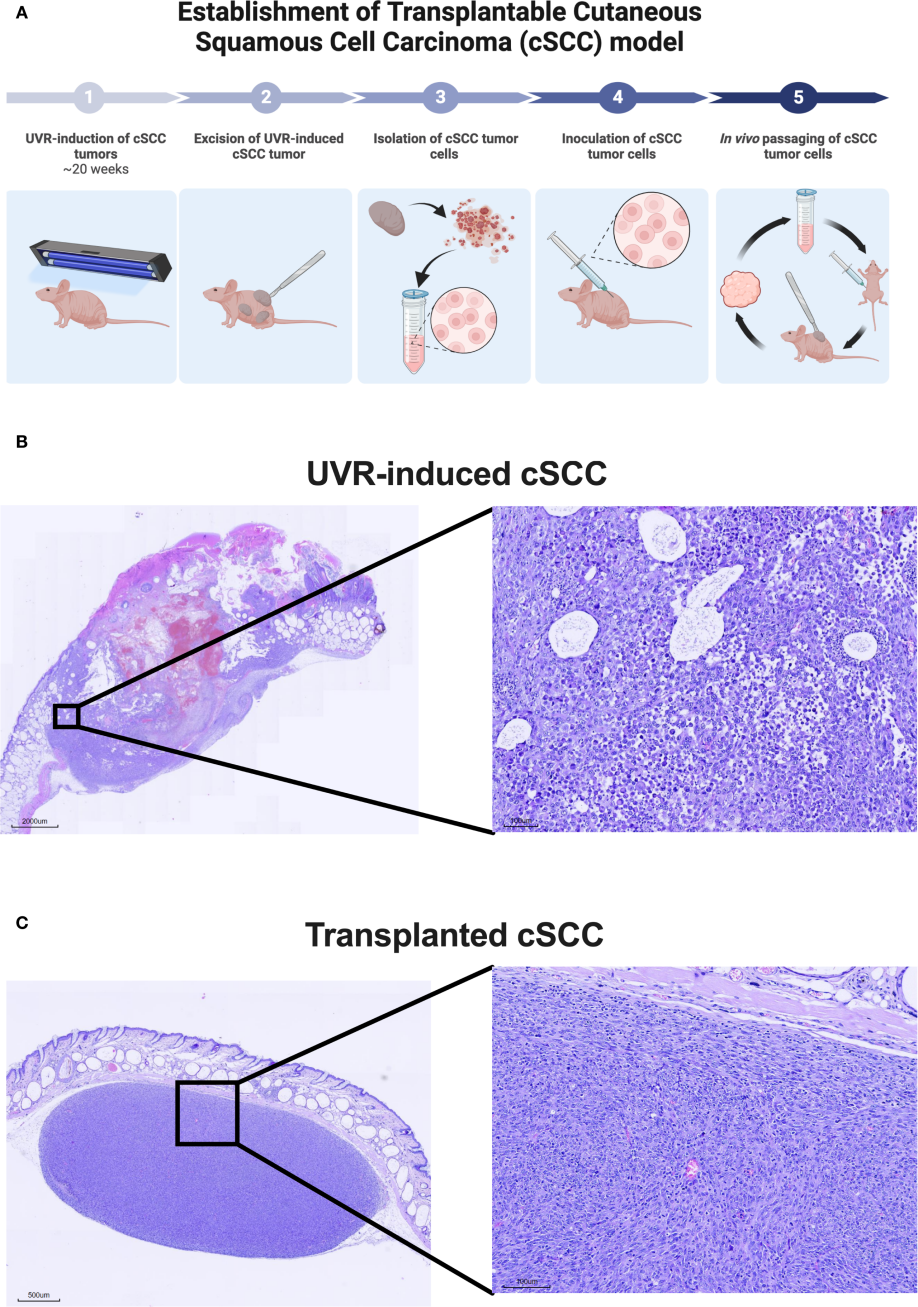
Establishment of syngeneic transplanted cutaneous squamous cell carcinoma (cSCC) mouse model. **(A)** Conceptual illustration of the establishment of the syngeneic transplanted cSCC tumor model based on tumor cells originating from the UVR-induced autochthonous cSCC tumor model. The model is established in the hairless and immunocompetent C3.Cg-*Hr^hr^*/TifBomTac mouse strain. **(B)** Representative histological image of UVR-induced cSCC tumor stained with hematoxylin and eosin-stained. **(C)** Representative histological image of a transplanted cSCC tumor used throughout this study stained with hematoxylin and eosin. The images show that transplanted cSCC tumors are located in the subcutaneous layer below *Panniculus carnosus*. Abbreviations: cSCC, cutaneous squamous cell carcinoma; UVR, ultraviolet radiation.

A syngeneic transplanted cSCC tumor model was established using tumor cells from the autochthonous UVR-induced cSCC tumor model (**Figure 2A**). The parental UVR-induced cSCC tumors were generated as previously described in Lerche et al (28). Briefly, mice were exposed to an erythema-inducing UVR-protocol thrice weekly. Mice developed superficial epidermally and dermally located cSCC tumors (**Figure 2B**) approximately 20 weeks after UVR initiation. UVR-induced tumors were aseptically excised and subsequently mechanically minced. Tumor matrix was further enzymatically dissociated in tubes containing Mouse Tumor Dissociation kit (Milteney Biotec, Bergisch Gladbach, Nordrhein-Westfalen, cat#130-096-730) for 1 hour at 37 °C in a Mini LabRoller™ tube rotator (LabNet International, Edison, NJ, USA). Enzyme digested tumor tissue was passed through 70 µm cell strainer (pluriSelect Life Science, Leipzig, Sachsen, Germany, cat# 43-10070) twice and washed with sterile 4 °C phosphate-buffered saline to obtain a single-cell solution of tumor cells. The tumor cells were counted using a Countess I™ (Thermo Fisher Scientific, Waltham, MA, USA) and diluted in Hank’s Balanced Salt Solution to a concentration of 20 x 10^6^ viable cells/mL. 2 x 10^6^ viable tumor cells were injected subcutaneously into the right flank of the mice. A single tumor established at the point of injection and no metastasis were observed throughout the study.

**Figure 3:**
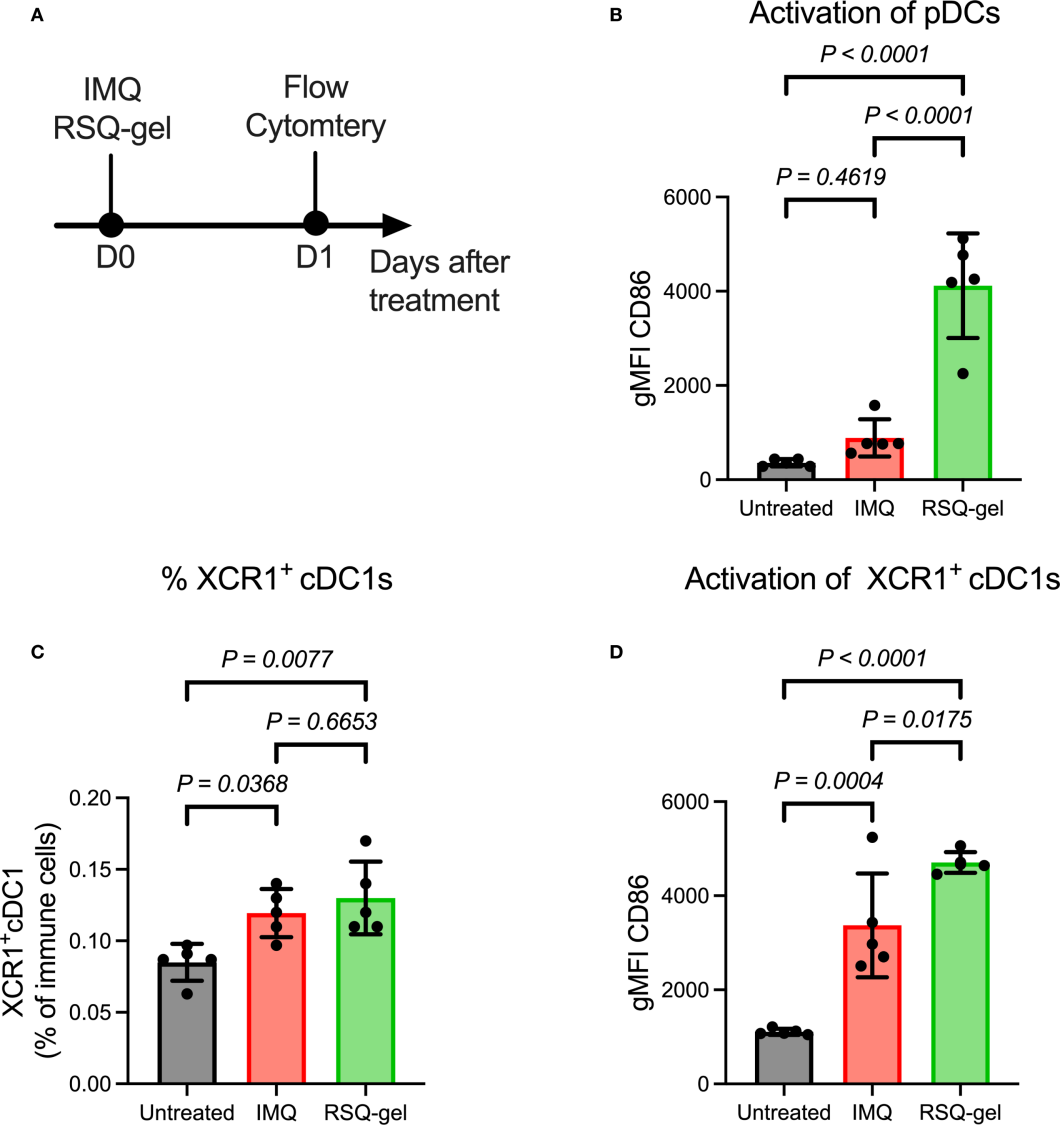
Intratumoral RSQ-gel treatment is associated with increased activation of plasmacytoid dendritic cells (pDC) and XCR1^+^ conventional type I dendritic cells (XCR1^+^ cDC1) in the tumor-draining lymph node. **(A)** Experimental timeline of *ex vivo* flow study in which tumor-draining lymph node were analyzed one day after treatment. **(B)** Geometric mean fluorescent intensity (gMFI) of CD86 on pDCs following treatment of either topical imiquimod (IMQ) or Intratumoral resiquimod in a sustained release gel (RSQ-gel). **(C)** Percentage XCR1^+^ cDC1s of all immune cells following treatment in the tumor-draining lymph node. **(D)** gMFI of CD86 on XCR1^+^ cDC1s following treatment. Sample size of n = 5 per group. One-way ANOVA with Tukey**’**s ***post hoc*** multiple comparison test was used for statistical testing. Abbreviations: cDC1, conventional type I dendritic cells; D, day; gMFI, geometric mean fluorescence intensity; IMQ, topical imiquimod cream; pDC, plasmacytoid dendritic cells; RSQ-gel, intratumoral injected sustained release formulated resiquimod gel; XCR1, X-C motif chemokine receptor 1.

**Figure 4:**
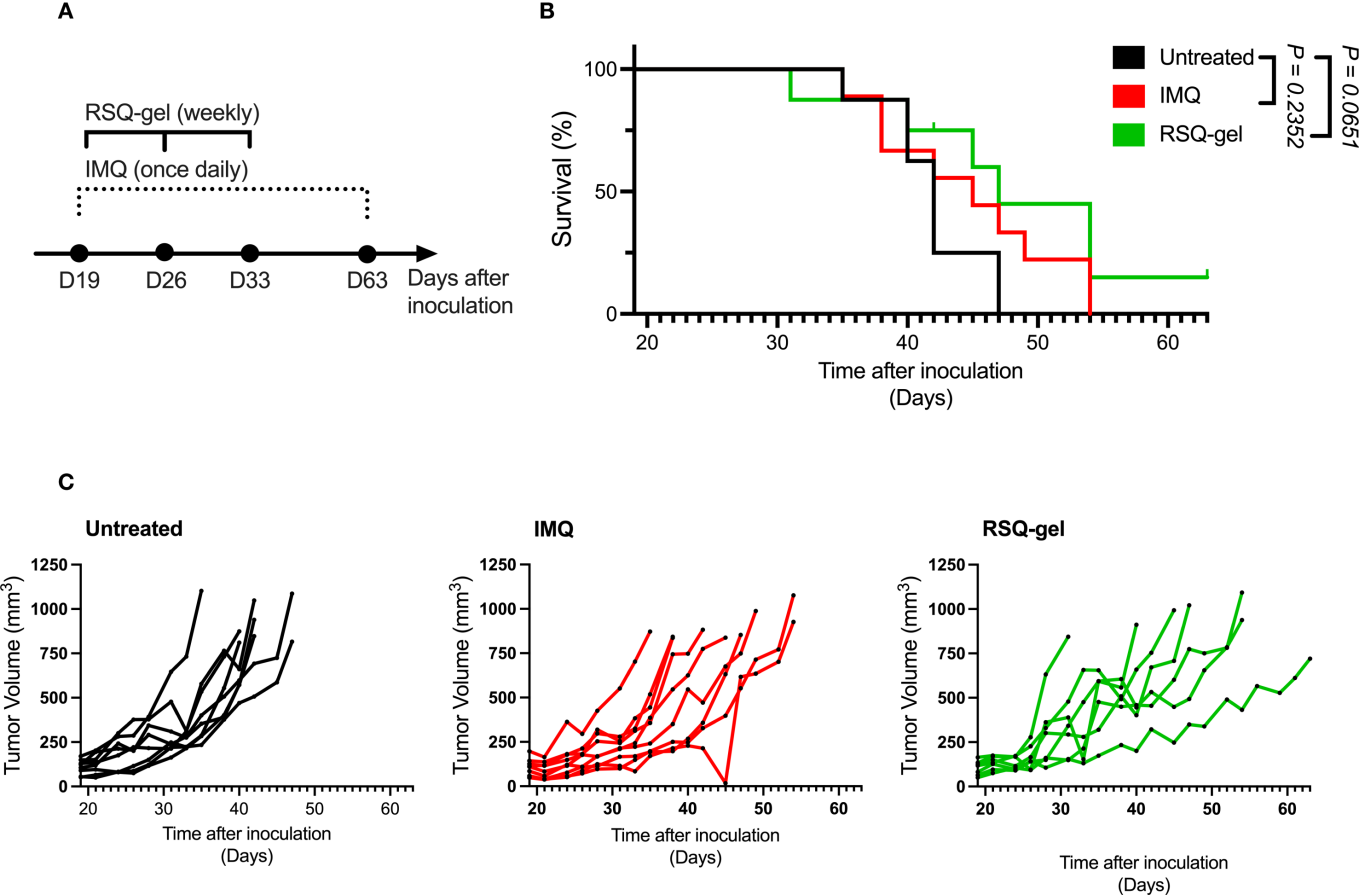
RSQ-gel and IMQ treatment delay tumor growth of some cSCC tumors. **(A)** Experimental timeline of daily imiquimod (IMQ) and weekly resiquimod-gel (RSQ-gel) treatments following tumor inoculation in cutaneous squamous cell carcinoma (cSCC) model. **(B)** Kaplan-Meier plot displaying percentage survival of cSCC mice over time after tumor cell inoculation. **(C)** Growth curves of each individual cSCC tumor within each group showing tumor volume (mm^3^) over days after tumor cell inoculation. Group sizes: Untreated (n = 8), IMQ (n = 9), RSQ-gel (n = 7). Log-rank test (Mantel-Cox) was used for statistical testing of survival. Abbreviations: D, day; IMQ, topical imiquimod cream; RSQ-gel, intratumoral injected sustained release formulated resiquimod gel.

**Figure 5:**
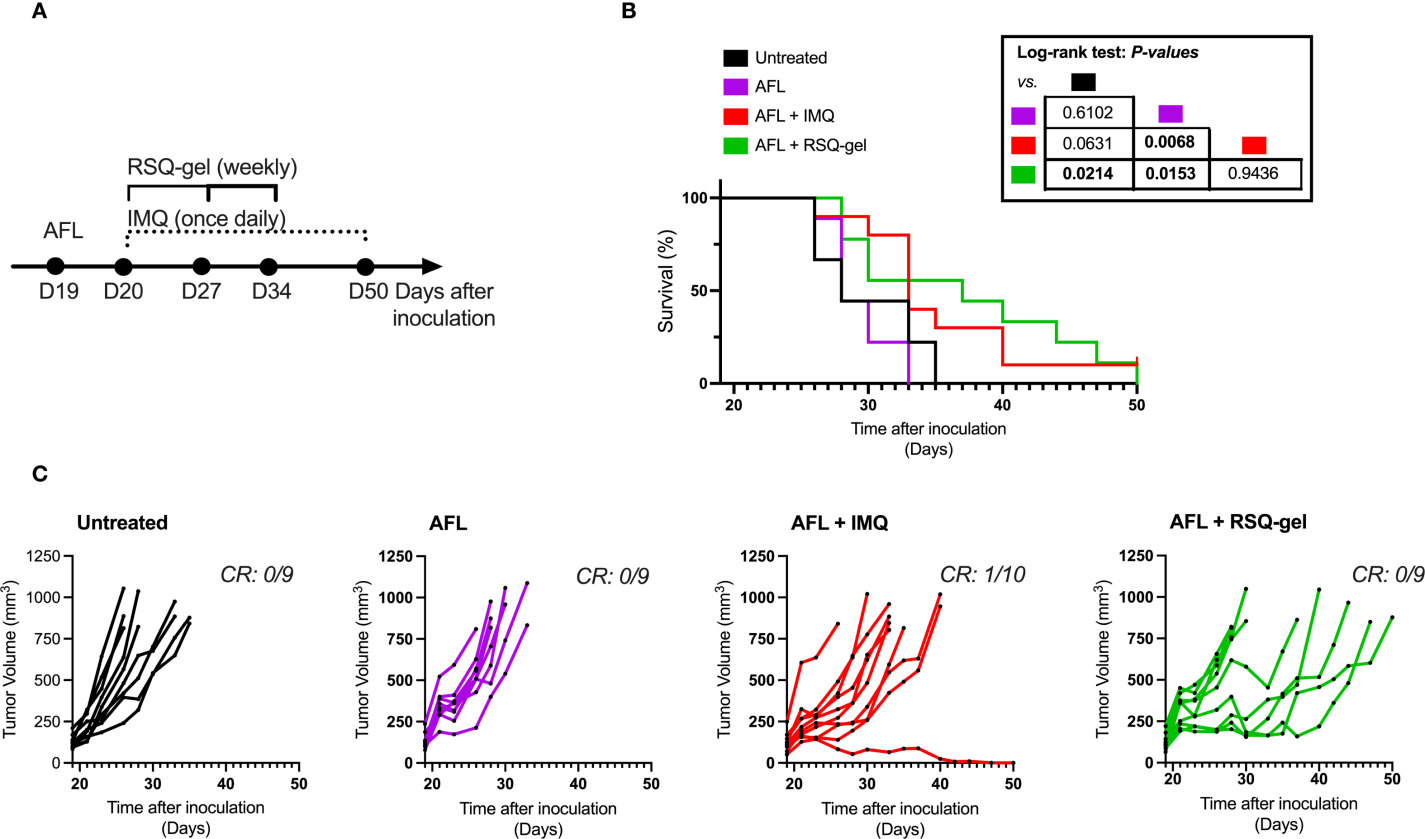
Antitumor efficacy of adjuvant ablative fractional laser (AFL) with RSQ-gel. **(A)** Experimental timeline describing time of treatment and dosing schedules following tumor inoculation. **(B)** Kaplan-Meier plot displaying percentage survival of cSCC mice over time after cutaneous squamous cell carcinoma (cSCC) tumor cell inoculation. **(C)** Growth curves of each individual cSCC tumor within each group showing tumor volume (mm^3^) over days after tumor cell inoculation. Number of complete responders (CR) of all subjects is indicated on each tumor growth plot. Group sizes: Untreated (n = *9*), *AFL (n = 9)*, *AFL+*IMQ (n = *10*), *AFL+*RSQ-gel (n = *9*). Log-rank test (Mantel-Cox) was used for statistical testing and p-values are indicated in the table located next to the graph. Abbreviations: AFL, ablative fractional laser; CR, complete responders; D, day; IMQ, topical imiquimod cream; RSQ-gel, intratumoral injected sustained release formulated resiquimod gel.

The tumor was excised once the length of the first generation of syngeneic transplanted cSCC tumor measured 12 mm in length. Tumor cell isolation and inoculation procedures were repeated as described above. Tumor cells were *in vivo* passaged for a total of four generations before use in flow cytometry and monotherapy studies presented in **Figures 3**, **4** and five generations for the combination therapy study presented in **Figure 5**. Both inoculations were conducted with tumor cells were derived from the same original UVR-induced cSCC tumor. The histology of the parental UVR-induced cSCC tumor and transplanted cSCC tumor are presented in **Figures 2B, C**.

### 2.4 Histology

Excised parental UVR-induced cSCC and transplanted cSCC tumors were fixed in 4% formalin and embedded in paraffin with a Shandon™ Excelsior ES^®^ (Thermo Fisher Scientific, Waltham, MA, USA) and sectioned at 4 µm with a Shandon Finesse Series Microtome (Thermo Fisher Scientific, Waltham, MA, USA) before transferred to a water bath at 42 °C and dried over night at 37 °C in a heating oven. Sections were deparaffinized, gradually hydrated. Sections were stained with Mayers hematoxylin for 5 minutes and washed in tap water followed by eosin-staining for 5 minutes. Slides were washed before dehydrated in graded ethanols. Airdried sections were mounted with Pertex. Slides were digitalized at 20x magnitude with a MoticEasyScan Pro (Motic, Xiamen, Fujian, China).

### 2.5 RSQ-gel formulation

Resiquimod (Ark Pharm. Inc., Wuhan, Hubei, China) was formulated in a gel as previously described in Jensen et al. (27). In short, sucrose benzoate, glyceryl trioctanoate and ethanol (60:25:15, w/w) were mixed by ultrasonication at 75 °C for 1.5–2 hours and vortexed until the gel matrix was transparent, and a homogenous solution was obtained. The gel matrix was mixed with resiquimod under magnetic stirring at 40-50 °C until resiquimod was fully dissolved. The final resiquimod concentration was 3 mg/mL. Formulated RSQ-gel was stored at 4 °C prior to administration. All chemicals for the gel matrix were purchased from Sigma Aldrich.

### 2.6 Treatment interventions

Each tumor in the RSQ-gel group was injected with 50 µL RSQ-gel corresponding to a dose of 7.5 mg/kg per mouse. RSQ-gel was injected in the center of the tumor with a 23G needle. Mice were anesthetized with isoflurane during RSQ-gel injections. RSQ-gel was administered once weekly for three weeks in the efficacy studies. IMQ treatment groups had IMQ 5% cream, topically applied on an area covering the tumor once daily throughout the study period. One sachet IMQ cream containing 12.5 mg of IMQ was used for treating 10 mice which corresponds to a dose of 62.5 mg/kg in mice. AFL treatment was given using a fractional CO_2_ laser Ultrapulse^®^ instrument equipped with DeepFX Handpiece (Lumenis Inc., San Jose, CA, USA). In combination studies, AFL treatment was given one day prior to IMQ or RSQ-gel treatment with the application settings: 10,600 nm wavelength, spot size 0.12 mm, treatment area 6 x 6 mm, 100 mJ/microbeam, pulse duration<1 ms), 5% density. Based on a previous study, this laser setting has been reported to achieve a penetration depth of approximately 1000 µm in murine skin (35).

### 2.7 Flow cytometry

The inguinal LN, draining the tumor, was isolated and passed through a 70 µm cell strainer to obtain a single-cell suspension. Each sample was transferred to a 96-well plate and washed with FACS buffer (0.5% bovine serum albumin and 0.1% sodium azide in phosphate‐buffered saline). Samples were incubated with 50 μg/ml purified anti‐mouse CD16/CD32 (BD Biosciences, San Jose, CA, USA cat#553142) for 5 minutes on ice to block Fc receptors prior to staining. Samples were stained for 30 minutes on ice protected from light with the antibody staining panel provided in **Supplementary Table S1** and subsequently washed three times in FACS buffer. Samples were analyzed on the LSRFortessa X‐20 Fortessa Flow Cytometer (BD Biosciences). To compensate for spectral spillover Ultra-Comp eBeads™ Plus Compensation Beads (Thermo Fisher Scientific, cat# 01‐3333) were single-stained with every antibody and ArC™ Amine Reactive Compensation Beads (Thermo-Fisher Scientific, cat# A10346) with viability dye and compensation matrix were calculated in FlowJo Software v10.10 (BD Biosciences). Data were analyzed with FlowJo Software v10.10. Gating strategies and fluorescence minus one-samples are shown in **Supplementary Figure S2**.

### 2.8 Visualization and statistics

Visualization of data and statistical analysis of experiments was performed with Prism v10 (GraphPad Software, https://www.graphpad.com/, San Diego, CA, USA). For statistical testing, one-way ANOVA with follow-up Tukey’s multiple comparison test was used for all flow experiments and log-rank test (Mantel-Cox) for survival analysis of Kaplan-Meier curves. A significance level of α = 0.05 was used throughout the studies. Prospective power calculations were performed to estimate group sizes in survival studies with 80% power, α = 0.05 and an effect size estimated based on previous experiments with RSQ-gel (27). Graphical illustrations were created using Biorender.com (BioRender, Toronto, ON, Canada).

## 3 Results

### 3.1 Intratumoral RSQ-gel recruits and activates cross-presenting dendritic cells in tumor-draining lymph node

It has been proposed that TLR7 agonists induce antitumor immune responses through activation of pDCs, facilitating activation of XCR1^+^ cDC1s that prime CD8^+^ T cells (16, 17, 19–22). The activation of pDCs was evaluated by measuring the expression of the activation marker CD86 24 hours post-treatment (**Figure 3A**). RSQ-gel treatment induced a substantial activation of pDCs compared with both IMQ treatment group (*P< 0.0001*) and untreated group (*P< 0.0001*), whereas the IMQ group showed no increased activation in comparison with the untreated group (*P = 0.4619*, **Figure 3B**).

The proportion of XCR1^+^ cDC1s among all immune cells in the tumor-draining LN was increased following RSQ-gel (*P = 0.0077*) and IMQ treatment (*P = 0.0368*), suggesting enhanced dendritic cell recruitment. There were no significant differences in the percentage of XCR1^+^ cDC1s between the treatments (*P = 0.6653*, **Figure 3C**). Further examination of the activation of XCR1^+^ cDC1s showed that RSQ-gel treatment led to a superior upregulation of CD86 on XCR1^+^ cDC1s compared to both IMQ treatment (*P = 0.0175*) and no treatment (*P< 0.0001*, **Figure 3D**).

### 3.2 RSQ-gel alone induces minimal delayed tumor growth in cSCC model

The activation of pDCs and XCR1^+^cDC1s encouraged further investigation of the antitumor efficacy of RSQ-gel. The RSQ-gel group received weekly intratumoral injection for three weeks, while IMQ group received daily topical IMQ throughout the study (**Figure 4A**).

Weekly RSQ-gel treatment did not result in a significant prolonged survival time compared to untreated controls in the cSCC model (*P = 0.0651*, **Figure 4B**). However, multiple cSCC tumors in RSQ-gel group showed delayed tumor growth compared to the untreated tumors (**Figure 4C**). RSQ-gel showed no significant differences in efficacy compared to daily topical IMQ treatment. No local or systemic toxicities were observed following RSQ-gel treatment. A transient weight loss was observed one day after the initial RSQ-gel treatment, but animals returned to body weights comparable to the control group within three days. Subsequent RSQ-gel treatment did not result in significant weight loss relative to control group (**Supplementary File**; **Supplementary Figure S1A**).

### 3.3 Adjuvant ablative fractional laser augments antitumor efficacy of RSQ-gel

Results from the monotherapy experiment demonstrated that RSQ-gel is inadequate in inducing significantly prolonged survival time in the cSCC model when administered as monotherapy. Consequently, we investigated whether combining RSQ-gel treatment with adjuvant AFL could improve the treatment efficacy in the cSCC model. Mice received a single AFL treatment one day prior to start of weekly RSQ-gel treatment or daily topical IMQ treatment (**Figure 5A**).

Adjuvant AFL with RSQ-gel treatment led to significant increased survival time compared to AFL alone (*P = 0.0153*) and untreated controls (*P = 0.0214).* AFL monotherapy did not improve survival time compared with untreated group (*P = 0.6102*). The efficacy of weekly RSQ-gel induced a similar antitumor efficacy as IMQ treatment with no significant differences in survival time between the groups (*P = 0.9436*). However, AFL prior to IMQ treatment only showed significant efficacy compared with AFL monotherapy (*P = 0.0068*) but not compared with untreated (*P = 0.0631*). **Figure 5B**). RSQ-gel treated cSCC tumors showed a delayed tumor growth compared to untreated (**Figure 5C**).

## 4 Discussion

This study demonstrates that intratumoral treatment with RSQ-gel combined with adjuvant AFL induces antitumor efficacy in a syngeneic transplanted cSCC model. RSQ-gel showed superior activation of pDCs and XCR1^+^cDC1s in the tumor-draining LN compared to topical IMQ treatment, highlighting the potent immunomodulatory properties of resiquimod. RSQ-gel with adjuvant AFL treatment resulted in delayed cSCC tumor growth and prolonged survival, whereas RSQ-gel as monotherapy only delayed growth of certain tumors. Weekly RSQ-gel achieved comparable efficacy to daily topical IMQ but requires fewer administrations and a lower administered dose.

TLR7 agonists are known to activate pDCs, triggering IFN-α secretion (36, 37). IFN-α secreted by activated pDCs promotes the recruitment and activation of cross-presenting dendritic cells, facilitating antitumor immunity through priming of CD8^+^ T-cells in the tumor-draining LN (16–18). In our study, RSQ-gel treatment increased the presence of cross-presenting XCR1^+^ cDC1s to the tumor-draining LN and showed superior activation of both pDCs and XCR1^+^ cDC1s compared to IMQ treatment. However, using CD86 alone as activation marker does not conclusively demonstrate functional activation. Further studies evaluating additional activation markers and cytokines are necessary to confirm the activation.

Both IMQ and RSQ-gel did not prolong survival in the cSCC model when given as monotherapies, although the tumor growth curves suggest delayed growth of several tumors. Resiquimod has previously shown significant antitumor efficacy in the CT26 colon carcinoma tumor model (27, 38). CT26 is considered a highly immunogenic tumor model due to its high mutational burden, high number of CD8^+^ cytotoxic T cells relative to regulatory T cells, and low infiltration of myeloid-derived suppressor cells (39, 40). CT26 demonstrates the highest reactivity to immune checkpoint inhibitor treatment across six common syngeneic cell-line derived tumor models (40). While the transplanted cSCC tumor in this study likely has a high mutational burden similar to the UVR-induced cSCC model (41), the transplanted cSCC may have a tumor immune microenvironment that is less sensitive to TLR7 agonists than CT26.

AFL is proposed to promote antitumor effects both by ablating tumor cells and indirectly by causing tissue injury, resulting in local infiltration of neutrophils, macrophages, and lymphocytes (30–33, 35, 42–45). Preclinical studies have shown that AFL can induce infiltration of tumor-specific CD8^+^ T cells in the CT26 tumor model, suggesting AFL treatment releases tumor-specific antigens (31, 32). In the current study, AFL treatment augmented improved survival time when combined with either RSQ-gel or IMQ treatment but did not induce delayed tumor growth or improved survival time as monotherapy, contrary to previous studies in the CT26 tumor model (31, 32). We hypothesize that while AFL alone is not sufficient to cause antitumor efficacy alone, it may synergize with TLR7 agonists in the cSCC model by promoting the release of tumor-specific antigens. These antigens may then be cross-presented to CD8^+^ T cells in the tumor-draining LN by TLR7 agonist-matured XCR1^+^ cDC1s, potentially resulting in the observed antitumor response. Functional assays evaluating antigen-specific T cells responses will be necessary to confirm whether AFL enhances cross-priming in the cSCC model.

A potential caveat of the used transplanted cSCC model is the subdermal localization of the tumor. AFL and IMQ, as monotherapies, has previously been described to significantly reduce tumor size in the parental UVR-induced cSCC model which is in contrast with the findings in the syngeneic transplanted cSCC model (34). The difference in antitumor efficacy of topical IMQ between the two studies may be explained by the dermal location of the UVR-induced cSCC tumor. Both AFL and topical IMQ likely penetrate deeper into epidermally located tumors in the UVR-induced cSCC model than a subcutaneously located tumor such as the syngeneic transplanted cSCC model.

Intratumoral administration of TLR7 agonists may be beneficial in human tumors due to improved bioavailability. The therapeutic potential of topical IMQ might be overestimated in mouse tumor models as it has been shown that while topical IMQ greatly penetrates mouse skin, it only has a limited penetration of pig skin which resembles human skin (46–48). By administering RSQ-gel intratumorally, this approach overcomes the challenges associated with skin penetration and thereby allows for broader drug distribution within tumors. In future studies, it is relevant to compare the drug concentration and distribution of RSQ-gel with topical IMQ in the cSCC tumor to identify whether RSQ-gel results in improved drug distribution in deeper tumor tissue. Despite these considerations, our findings demonstrate that RSQ-gel results in comparable antitumor efficacy to topical IMQ in the transplanted cSCC model but with a lower drug dose and fewer administrations, potentially leading to higher treatment compliance compared to daily IMQ application. A limitation of mouse models is that, while resiquimod activates both TLR7 and TLR8 in humans, only TLR7 is activated in mice by RSQ (11, 49, 50).

In summary, we demonstrate that weekly intratumoral treatment with resiquimod in a sustained release gel, RSQ-gel, with adjuvant AFL generates a significant antitumor efficacy in a syngeneic transplanted cSCC model. The RSQ-gel efficacy was comparable to daily topical IMQ cream. Together, RSQ-gel with adjuvant AFL may offer a novel approach to cSCC lesions.

## Data Availability

The raw data supporting the conclusions of this article will be made available by the authors, without undue reservation.
