# Supplementary figures and images for: Intratumoral sustained release of resiquimod with ablative fractional laser induces efficacy in a cutaneous squamous cell carcinoma mouse model
Source: Front Immunol. 2025 Oct 8;16:1625867. doi: 10.3389/fimmu.2025.1625867 (PMC12540399; doi:10.3389/fimmu.2025.1625867)

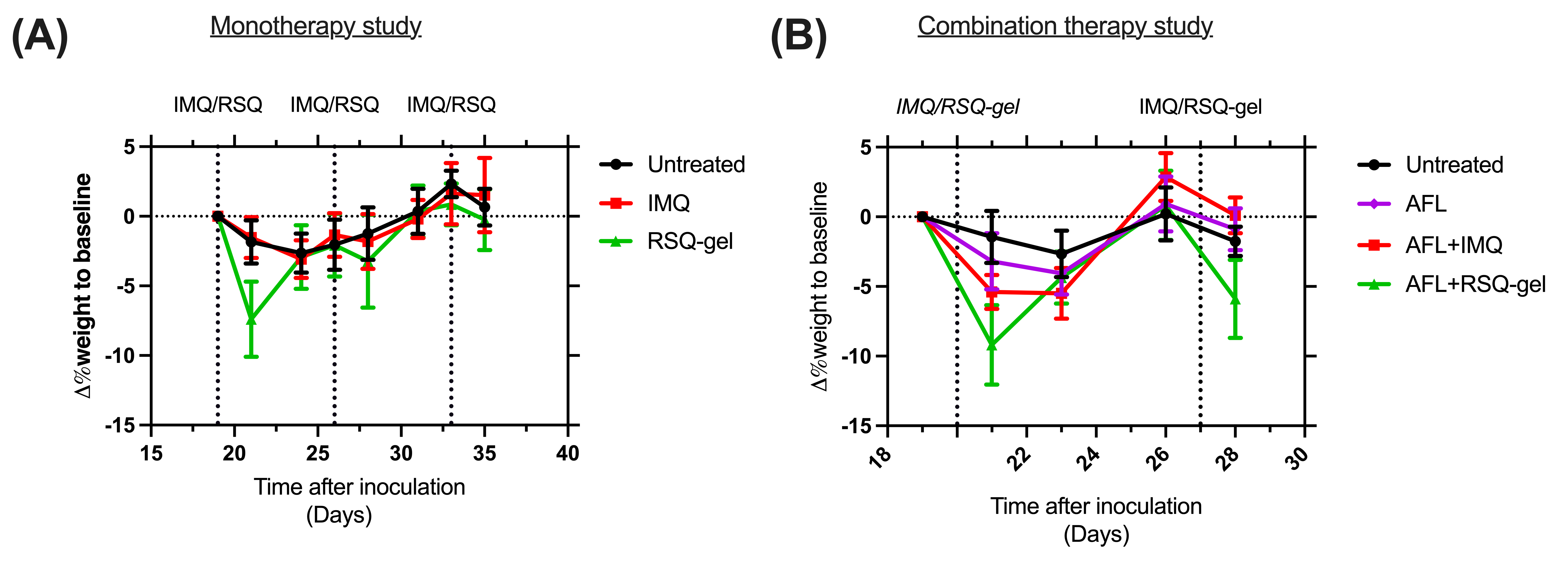

Supplement: Supplementary file 1 [file Image1.tiff]

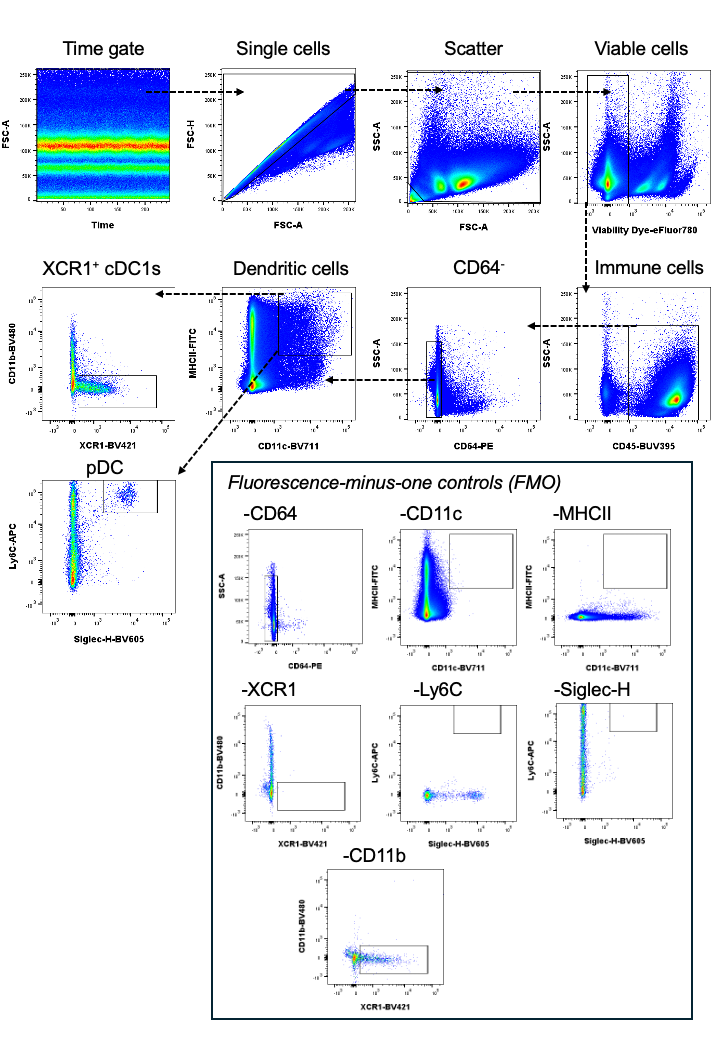

Supplement: Supplementary file 2 [file Image2.tiff]
