# Supplementary material for: Intratumoral sustained release of resiquimod with ablative fractional laser induces efficacy in a cutaneous squamous cell carcinoma mouse model
Source: Front Immunol. 2025 Oct 8;16:1625867. doi: 10.3389/fimmu.2025.1625867 (PMC12540399; doi:10.3389/fimmu.2025.1625867)
Supplement: Supplementary file 3 [file Table1.docx]

| ***Antigen*** | ***Clone*** | ***Fluorophore*** | ***Dilution*** | ***Supplier*** | ***Catalogue no.*** |
| --- | --- | --- | --- | --- | --- |
| CD45 | 30-F11 | BUV395 | 1:200 | BD Biosciences | 564279 |
| CD8a | 53-6.7 | BUV737 | 1:100 | BD Biosciences | 612759 |
| XCR1 | ZET | BV421 | 1:200 | BioLegend | 148216 |
| CD11b | M1/70 | BV480 | 1:400 | BD Biosciences | 566149 |
| Siglec-H | 440c | BV605 | 1:200 | BD Biosciences | 747673 |
| CD11c | N418 | BV711 | 1:100 | BioLegend | 117349 |
| I-A/I-E (MHCII) | M5/114.15.2 | FITC | 1:200 | BioLegend | 107606 |
| CD64 | X54-5/7.1 | PE | 1:50 | BioLegend | 139304 |
| CD86 | GL1 | PE-Cy7 | 1:100 | BD Biosciences | 560582 |
| Ly6C | HK1.4 | APC | 1:100 | Thermo Fisher Scientific | 17-5932-82 |
| *Dead cells*^‡^ | N/A | eFluor 780 | 1:500 | Thermo Fisher Scientific | 65-0865 |
| ‡Viability dye used to discriminate dead cells from viable cells. | | | | | |

**Table S1** Antibodies and viability dye for flow cytometry analysis.
